# Donor age over 55 is associated with worse outcome in lung transplant recipients with idiopathic pulmonary fibrosis

**DOI:** 10.1186/s12890-024-03317-x

**Published:** 2024-10-09

**Authors:** Isabelle Moneke, Ecem Deniz Ogutur, Anastasiya Kornyeva, Sebastian Fähndrich, David Schibilsky, Sibylle Bierbaum, Martin Czerny, Daiana Stolz, Bernward Passlick, Wolfgang Jungraithmayr, Bjoern Christian Frye

**Affiliations:** 1 Faculty of Medicine, Department of Thoracic Surgery, Medical Center, University of Freiburg, Hugstetter Str. 55, 79106 Freiburg, Germany; 2 Faculty of Medicine, Department of Pneumology Medical Center, University of Freiburg, Freiburg, Germany; 3 Faculty of Medicine, Clinic for Cardiovascular Surgery, University Heart Centre Freiburg – Bad Krozingen, University of Freiburg, Freiburg, Germany; 4 Faculty of Medicine, Institute of Virology, Medical Center, University of Freiburg, Freiburg, Germany; 5 Department of Thoracic Surgery, University Hospital Zurich, Zurich, Switzerland

**Keywords:** Donor age, Lung transplantation, Idiopathic pulmonary fibrosis, Organ selection

## Abstract

**Background:**

Lung transplantation (LTx) remains the only efficient treatment for selected patients with end-stage pulmonary disease. The age limit for the acceptance of donor organs in LTx is still a matter of debate. We here analyze the impact of donor organ age and the underlying pulmonary disease on short- and long-term outcome and survival after LTx.

**Methods:**

Donor and recipient characteristics of LTx recipients at our institution between 03/2003 and 12/2021 were analyzed. Statistical analysis was performed using SPSS and GraphPad software.

**Results:**

In 230 patients analyzed, donor age ≥ 55 years was associated with a higher incidence of severe primary graft dysfunction (PGD2/3) (46% vs. 31%, *p* = 0.03) and reduced long-term survival after LTx (1-, 5- and 10-year survival: 75%, 54%, 37% vs. 84%, 76%, 69%, *p* = 0.006). Notably, this was only significant in recipients with idiopathic pulmonary fibrosis (IPF) (PGD: 65%, vs. 37%, *p* = 0.016; 1-, 5-, and 10-year survival: 62%, 38%, 16% vs. 80%, 76%, 70%, *p* = 0.0002 respectively). In patients with chronic obstructive pulmonary disease (COPD), donor age had no impact on the incidence of PGD2/3 or survival (21% vs. 27%, *p* = 0.60 and 68% vs. 72%; *p* = 0.90 respectively). Moreover, we found higher *Torque-teno* virus (TTV)-DNA levels after LTx in patients with IPF compared to COPD (X^2^ = 4.57, *p* = 0.033). Donor age ≥ 55 is an independent risk factor for reduced survival in the whole cohort and patients with IPF specifically.

**Conclusions:**

In recipients with IPF, donor organ age ≥ 55 years was associated with a higher incidence of PGD2/3 and reduced survival after LTx. The underlying pulmonary disease may thus be a relevant factor for postoperative graft function and survival.

**Trial registration number DKRS:**

DRKS00033312.

**Supplementary Information:**

The online version contains supplementary material available at 10.1186/s12890-024-03317-x.

## Introduction

For selected patients with end-stage chronic lung disease, lung transplantation is an established and effective treatment option [1]. Although survival rates have improved over the last decade, they are still lower compared to other solid organ transplantations due to a high number of complications, e.g., recurrent infection [1] or the development of chronic lung allograft dysfunction (CLAD) [2–4].

The 2013 report of the Registry of the International Society for Heart and Lung Transplantation shows an increase in the median age of lung transplant recipients from 45 to 55 years over the preceding decade [5]. This may increase morbidity and mortality after LTx since older patients are more likely to receive an organ [6]. The shortage of donor organs is a persistent problem resulting in a waiting list mortality of approximately 10–13% [3]. Therefore, not only the characteristics of the recipient are subject to discussion [7, 8], but also how to expand the donor pool and find the best possible match for the respective patient on the waiting list [9].

The debate on organ selection remains controversial regarding the use of older organs for transplantation [10, 11]. In previous studies, the ‘ideal donor’ is described as < 55 years of age [9, 10, 12], However, this statement does no longer reflect current practice and over the last decade the use of donors ≥ 55 years of age has been divergently handled by many transplant centres [10, 13–15].

Particularly the impact of the recipient’s underlying pulmonary disease on short and long-term outcome is incompletely understood. Fibrotic and obstructive lung diseases differ not only morphologically, but also regarding the underlying pathophysiology including different immunological alterations. Aside from idiopathic pulmonary fibrosis (IPF), there are many other causes of pulmonary fibrosis such as autoimmune/connective tissue diseases, e.g., rheumatoid arthritis, environmental exposures to coal or silica, or allergens, e.g., bird fancier’s lung [16, 17]. The pathogenesis of IPF is multifactorial, involving various processes that lead to dysregulation in wound healing with widespread scarring of the lungs [16]. It is considered an age-related disease, which usually occurs in patients older than 50 years, and is increasing in both, incidence, and prevalence [18, 19]. Moreover, altered innate and adaptive immune cell responses have both been linked to myofibroblast biology and fibrogenesis [18] and thus to fibrotic lung disease. In the context of lung transplantation, a dysregulated immune system might pose an additional challenge for the postoperative course and long-term survival. Monitoring immunosuppression after lung transplantation is challenging, and drug blood levels alone are insufficient to determine the individual status of immunosuppression [20, 21].

This study aims to analyse the impact of donor age, as well as the impact of the underlying pulmonary diseaseon graft function and short- and long-term outcome in our lung transplant cohort.

## Methods

### Design and study population

We identified all patients who underwent lung transplantation at the Department of Thoracic Surgery, Medical Centre – University of Freiburg between March 2003 and December 2021. Complete follow-up was available for 91% of the patients. Characteristics of recipients and donors were retrospectively analysed. Patients who underwent en-bloc heart-lung transplantation were excluded (Supplementary Figure S1). Most patients suffer from IPF or COPD as an underlying pulmonary disease. A detailed distribution is shown in Supplementary Figure S2. All patients were regularly followed up at our transplant outpatient centre. Basiliximab was used for induction therapy and standard immunosuppression consisted of prednisone, mycophenolate mofetil and tacrolimus, with adaptation depending on multiple factors, e.g., kidney function.

Moreover, all patients received the standard posttransplant antibiotic prophylaxis, CMV prophylaxis and antifungal prophylaxis. In case the donor and recipient were CMV negative, CMV prophylaxis was provided with Aciclovir for 3 months. Ganciclovir was given for 6 months in case both, or only the recipient, were CMV positive, and for 9 months in case of a high-risk constellation with the donor being positive and the recipient negative for CMV.

Recipient data, e.g., lung function analysis, bronchoscopic biopsy results as well as blood tests and clinical examination results were collected by checking electronic medical records, discharge reports and autopsy reports. Donor data were taken from the donor reports provided by Eurotransplant.

### Definitions

The predicted total lung capacity (pTLC) of the donor was calculated according to the formula provided by the Eurotransplant 2013 annual report. For male donors: 7.99x(height) – 7.08 and for female donors: 6.60x (height) – 5.7 [22].

Primary graft dysfunction (PGD) was defined according to the ISHLT criteria based on the PaO_2_/F_i_O_2_ ratio and the presence of diffuse parenchymal infiltrates in the allograft on the chest radiograph within 72 h [23]. All patients were evaluated according to these criteria regardless of the time of transplantation. PGD grade 2 and higher (PGD2/3) was considered relevant for the clinical outcome.

#### *Torque-Teno virus* (TTV) copy numbers

TTV copy number kinetics after lung transplantation from a previous study were re-analyzed [24]. *Torque-teno virus* (TTV) copy numbers in plasma samples of 33 patients (22 patients with underlying idiopathic pulmonary fibrosis (IPF) and 11 patients with COPD) were compared. Samples were collected pre-transplant and every following month for up to one year after lung transplantation.

### CMV reactivation

The CMV reactivation cut-off was defined as > 3000 copies/mL after the prophylaxis had ended, similar to other studies [25, 26].

### Statistical analysis

To estimate overall survival, we applied the Kaplan-Meier method. The log-rank test was used for comparison of the survival curves of the lung transplant recipients. Fischer’s exact test was used to analyse connections between different parameters in patients with and without underlying fibrotic lung disease. To analyse predictors of survival univariate and multivariate Cox proportional hazard regression analysis was used. To estimate the the difference in TTV load between patients with COPD and IPF a non-parametric Friedman Test was applied. All tests were two-tailed and a p-value < 0.05 was considered statistically significant. SPSS (Version 27, IBM Corporation, New York, NY, USA) and GraphPad Prism (Version 9, GraphPad Software, San Diego, CA 92108, USA) were used for all statistical analyses.

## Results

**Fig. 1:**
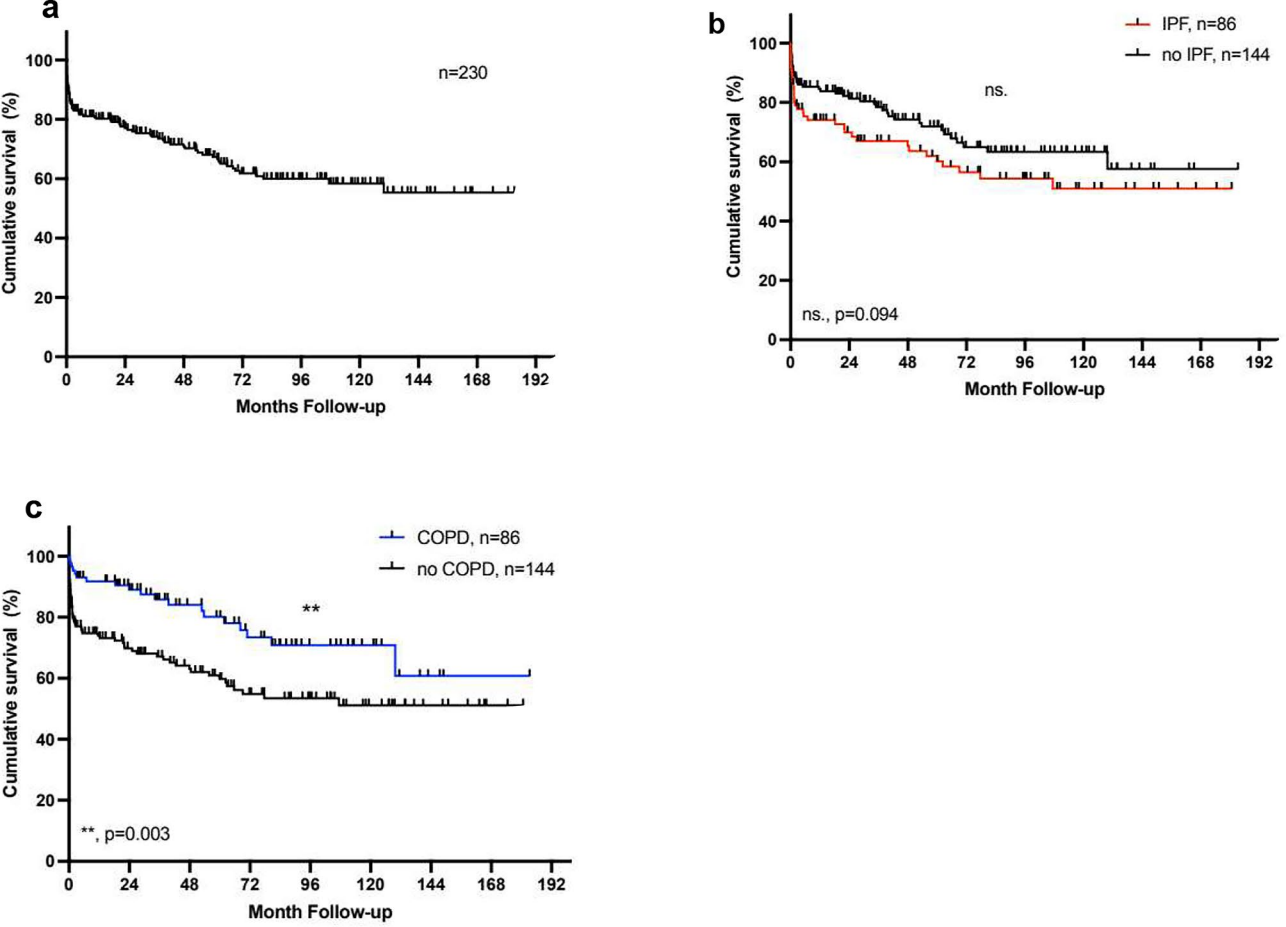
Kaplan-Meier Analysis of survival after lung transplantation. **a**) Overall post-transplant survival: 1-, 5- and 10-year survival: 80%, 67% and 58%. **b**) Survival with IPF: 1-, 5-, and 10-year survival 74%, 60%, and 51% vs. 91%, 75%, 66%. **c**) Survival with COPD: 1-,5- and 10-year survival 91%,80% and 70% vs. 74%, 60% and 51%. The log-rank test was used for the comparison of the survival curves

230 patients (120 male, 110 female) underwent lung transplantation at our institution between March 2003 and December 2021. 203 (88%) patients had a double lung transplantation, and 27 (12%) patients had a single lung transplantation. The overall 1-, 5- and 10-year-survival rate was 80%, 67% and 58% respectively (Fig. 1A). Basic donor and recipient characteristics are shown in Table 1.

**Table 1:** Basic patient characteristics of donors and recipients

Variable	Recipients				Corresponding Donors			
	Overall (*n* = 230)	IPF (*n* = 86)	COPD (*n* = 86)	Other (*n* = 58)				
Sex								
Male	118 (51%)	53 (38%)	41 (48%)	24 (43%)	112 (49%)	33 (62%)	52 (60%)	27 (47%)
Female	112 (49%)	33 (62%)	45 (52%)	34 (57%)	118 (51%)	53 (38%)	34 (40%)	31 (53%)
Age at LTX (years)								
Min Max Median	17 69 58	36 69 59	46 67 60	17 65 58	13 74 51	14 74 51	15 73 52	13 70 51
Age groups (years)								
< 18 18–29 30–39 40–49 50–59 60–69 > 69	1 (0.5%) 9 (4%) 7 (3%) 25 (11%) 91 (40%) 98 (43%) 0 (0%)	0 (0%) 0 (0%) 2 (2%) 11 (13%) 34 (40%) 39 (45%) 0 (0%)	0 (0%) 0 (0%) 0 (0%) 4 (5%) 37 (43%) 45 (52%) 0 (0%)	1 (2%) 9 (16%) 5 (9%) 10 (17%) 20 (34%) 14 (29%) 0 (0%)	10 (04%) 18 (08%) 26 (11%) 54 (23%) 61 (27%) 49 (21%) 12 (05%)	2 (2%) 8 (9%) 12 (14%) 19 (22%) 18 (21%) 22 (26%) 5 (6%)	3 (3%) 8 (9%) 11(13%) 19 (22%) 20 (23%) 20 (23%) 5 (6%)	5 (9%) 2 (3%) 3 (5%) 16 (28%) 23 (40%) 7 (12%) 2 (3%)
BMI at LTX (kg/m^2^)								
Min	14.8	14.8	15.9	15.2	14.2	14.1	18.5	16.3
Max	33.7	33.1	31.1	33.7	46.9	46.9	39.1	34.7
Median	23.1	24.2	22.1	23	24.8	24.2	25.4	24.8
LAS at LTX								
Min	31	32	31	32				
Max	96	95	83	96				
Median	39	45	34	39				
Pre LAS*	69 (30%)	32(37%)	21(24%)	16 (28%)				
HU	35	19	4	12				
U	24	9	12	3				
T	7	2	5	0				
Donor > 55 (%)								
LAS era					80 (50%)	27 (50%)	31 (48%)	22 (40%)
Pre-LAS era					13 (19%)	7 (20%)	2 (10%)	4 (7%)
Comorbidity								
CHD	38 (17%)	12 (14%)	18 (21%)	8 (14%)				
aHT	66 (29%)	27 (31%)	33 (38%)	6 (10%)				
DM	44(19%)	17 (20%)	15 (17%)	12 (21%)				
Comorbidity Donor								
CHD Smoker Median MV (days) Median pO_2_ (mmHg)					34 (15%) 75 (33%) 3 447	8 (9%) 25 (29%) 3 460	8 (9%) 32 (37%) 3 440	18 (31%) 18 (31%) 3 450
Operation								
DLTx SLTx ECMO Intraop. only Intra- + postop. Bridging	203 (88%) 27 (12%) 82 (36%) 70 (85%) 9 (11%) 3 (4%)	74 (86%) 12 (14%) 43 (50%) 36 (84%) 6 (14%) 1(2%)	73 (85%) 13 (15%) 13 (15%) 12 (92%) 1(8%) 0(0%)	56 (97%) 2 (3%) 26 (49%) 22 (38%) 2 (3%) 2 (3%)				

Basic characteristics of donors and recipients. BMI = body Mass Index, COPD = chronic obstructive pulmonary disease, IPF = idiopathic pulmonary fibrosis, EAA = exogenous allergic alveolitis, AAT = alpha-1 antitrypsin deficiency, CHD = coronary heart disease. * in the pre-LAS era, there was no information on the HU/U/T Status for 2 patients in the IPF group

**Table 2:** Univariate and multivariate Cox proportional hazard regression analysis to identify predictors of survival

Variables (*n* = 230)	Univariate, HR (95% CI)	*p*	Multivariate, HR (95% CI)	*p*
Sex				
Gender (female)	1.653 (1.052–2.597)	**0.046**	1.247(0.732–2.126)	0.42
Underlying disease				
IPF COPD	1.463 (0.919–2.327) 0.572 (0.365–0.896)	0.094 **0.003**	1.489 (0.847–2.719)	0.12
CLAD*				
All patients IPF COPD	2.399 (1.154–4.990) 1.824 (0.581–7.924) 1.741 (0.512–5.917)	**0.019** 0.24 0.31	1.076 (0.585–1.934)	0.84
PGD2/3				
All patients IPF COPD	3.225 (1.985–5.239) 2.202 (1.146–4.234) 3.757 (1.511–9.345)	< **0.0001** **0.0124** **0.017**	2.324 (1.447–3.752) 1.808 (0.897–3.744) 0.696 (0.331–1.320)	**0.0005** 0.10 0.30
Recipient ≥ 55 years				
All patients IPF COPD	0.865 (0.539–1.386) 1.114 (0.546–2.273) 0.721 (0.255–2.037)	0.55 0.77 0.50		
Donor ≥ 55 years				
All patients IPF COPD	2.277 (1.420–3.660) 3.311 (1.646–6.663) 1.065 (0.400 2.834)	**0.0006** **0.0002** 0.90	1.817 (1.115–2.975) 2.969 (1.431–6.380)	**0.017** **0.004**
Recipient comorbidity				
Diabetes mellitus CHD	1.295 (0.735–2.282) 0.903 (0.591–1.378)	0.37 0.64		
Donor comorbidity				
Nicotine CHD	0.997 (0.624–1.593) 1.576 (0.751–3.308)	0.99 0.23		
Sex				
Surgery SLTX All patients				
COPD IPF ECMO	1.016 (0.520–1.983) 1.033 (0.297–3.597) 1.246 (0.514–3.021)	0.96 0.96 0.60		
All patients COPD IPF	2.480 (1.559–3.943) 3.063 (0.898–10.44) 1.405 (0.731–2.699)	**< 0.0001** **0.013** 0.31	2.036 (1.245–3.378) 1.145 (0.542–2.190)	**0.005** 0.70

Predictors of survival. IPF = idiopathic pulmonary fibrosis, COPD = chronic obstructive pulmonary disease, ECMO = extracorporeal membrane oxygenation, CHD = coronary heart disease, CLAD = chronic lung allograft dysfunction, *includes all patients that survived at least 6 months. If not mentioned otherwise, the values are calculated for all patients. The information on PGD is missing for one patient

**Fig. 2:**
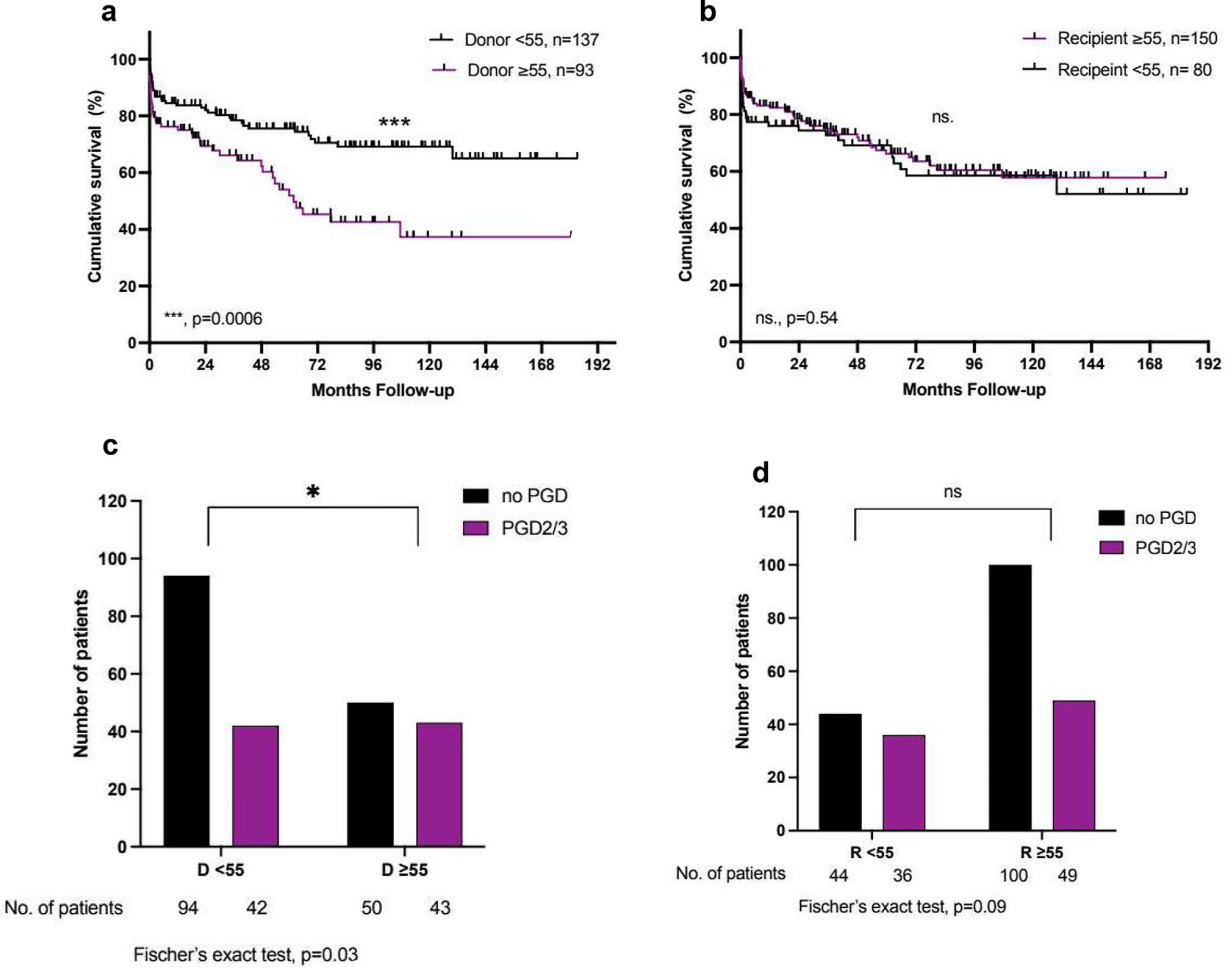
**a** + **b**: Kaplan-Meier Analysis of survival after lung transplantation: for donors and recipients < 55 and ≥ 55 years of age. **c** + **d**: Incidence of primary graft dysfunction grade 2 or 3 in donors and recipients ≥ 55 years of age. For one patient there is no information available on PGD

In the whole study cohort, a donor age of ≥ 55 years at the time of LTx was associated with reduced overall survival (1-, 5- and 10-year survival: 75%, 54%, 37% vs. 84%, 76%, 69%; *p* = 0.0006) and an increased incidence of PGD 2/3 (46% vs. 31%, *p* = 0.03) (Fig. 2). The significantly reduced survival was consistent for donor lungs ≥ 60 and ≥ 65 years and the also incidence of PGD2/3 was increased for donors ≥ 60 compared to younger donors (Supplementary Figure S3). PGD2/3 and donor age ≥ 55 years were found to be independent predictors of reduced survival in the whole cohort in multivariate analysis (*p* = 0.0005 and *p* = 0.017 respectively) (Table 2). There was no statistically significant impact of donor age on the development of CLAD (*p* = 0.28), however, CLAD was identified as a predictor of reduced survival in univariate analysis (*p* = 0.019). Interestingly, the recipient’s age at the time of LTx did not correlate with the incidence of PGD or long-term survival in either group.

**Fig. 3:**
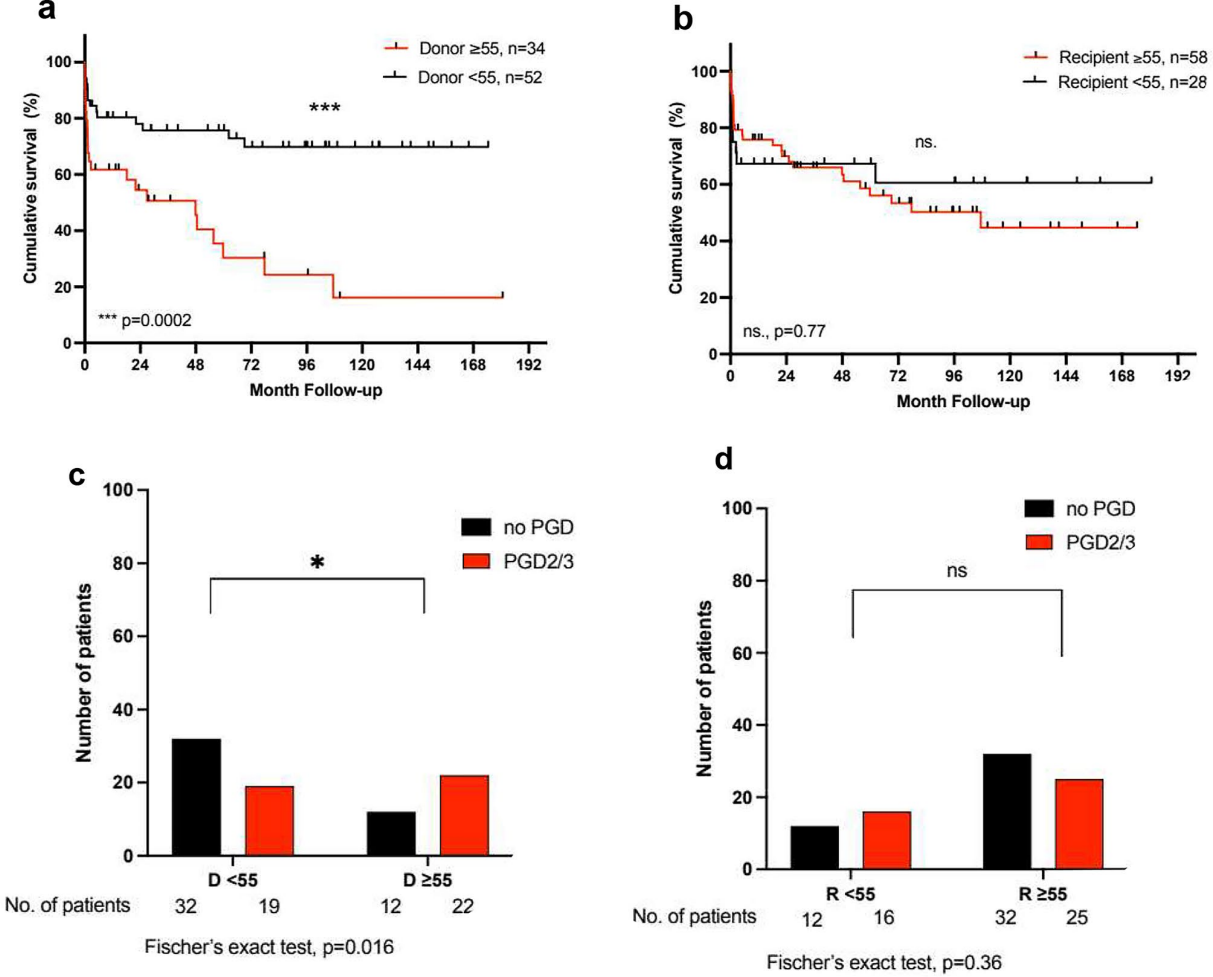
**a** + **b**: Kaplan-Meier Analysis of survival after lung transplantation in patients with IPF for donors and recipients < 55 and ≥ 55 years of age. **c** + **d**: Incidence of primary graft dysfunction grade 2 or 3 PGD2/3 in patients with IPF for donors and recipients < 55 and ≥ 55 years of age. The log-rank test was used for the comparison of the survival curves. For one patient there is no information available on PGD. D = donor, R = recipient

**Fig. 4:**
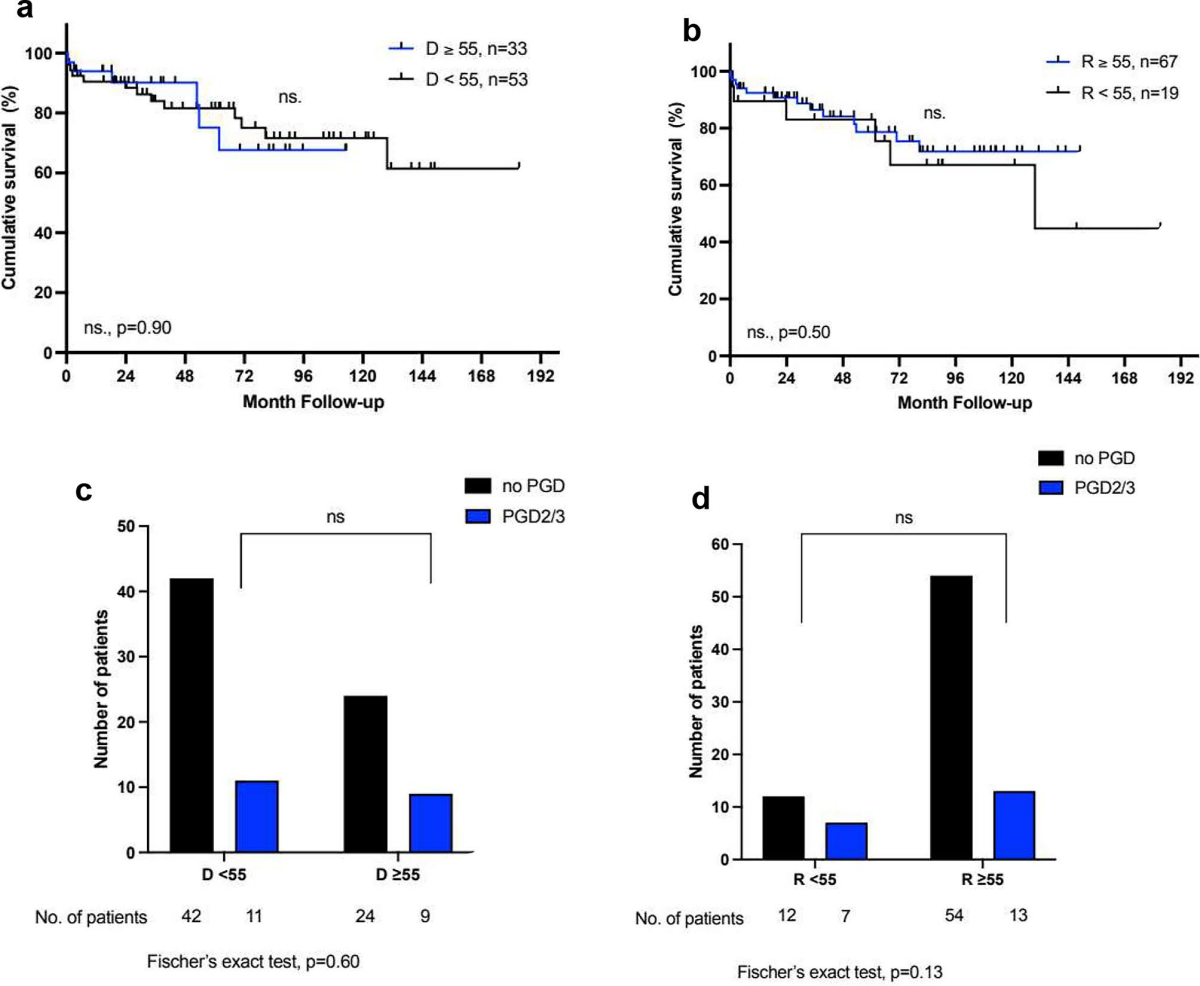
**a** + **b**: Kaplan-Meier Analysis of survival after lung transplantation in patients with COPD for donors and recipients < 55 and ≥ 55 years of age; **c** + **d**: Incidence of primary graft dysfunction grade 2 or 3 (PGD2/3 ) in patients with COPD for donors and recipients < 55 and ≥ 55 years of age. The log-rank test was used for the comparison of the survival curves. For one patient there is no information available on PGD. D = donor, R = recipient

In the next step, we analysed if there was a differential impact of donor age depending on the recipients’ underlying pulmonary disease. We identified 86 (37%) patients with idiopathic pulmonary fibrosis (IPF) and 86 (37%) patients with chronic obstructive pulmonary disease (COPD). Basic donor and recipient characteristics were comparable in both groups (Table 1). In general, overall survival after LTx was similar in patients with IPF (*p* = 0.094) and increased in patients with COPD (*p* = 0.003) (Fig. 1B + C). Notably, only in patients with IPF, a donor age ≥ 55 years was associated with a higher incidence of severe primary graft dysfunction (PGD2/3) (65% vs. 37%; *p* = 0.016) and with a considerably reduced long-term survival (1-, 5-, and 10-year survival: 62%, 38%, 16% vs. 80%, 76%, 70%; *p* = 0.0002) (Fig. 3). On the other hand, a donor age ≥ 55 had no impact on survival nor the incidence of PGD2/3 in patients with COPD (68% vs. 72%, *p* = 0.90 and 21% vs. 27%, *p* = 0.60 respectively) (Fig. 4). Again, the older age of the recipients was not associated with reduced overall survival or the incidence of PGD2/3 in either group. For patients with IPF, a donor age ≥ 55 years was identified as an independent predictor of reduced survival in multivariate Cox regression analysis (*p* = 0.004) (Table 2). The development of CLAD was not affected by donor or recipient age in either subgroup. As expected, the median LAS score was higher in the IPF group, however, there was no significant difference in the percentage of donor organs > 55 years between the groups in the LAS era (Table 1).

Single lung transplantation was performed in 12 (14%) patients with IPF and 13 (15%) patients with COPD. There was no difference in survival in either group compared to patients who underwent double lung transplantation. While more patients in the IPF subgroup were transplanted on ECMO support (43/86), there was no statistically significant association between the use of perioperative ECMO and survival (*p* = 0.31), unlike in the COPD subgroup (*p* = 0.013) and the whole cohort (p = < 0.0001) (Table 2).

**Fig. 5:**
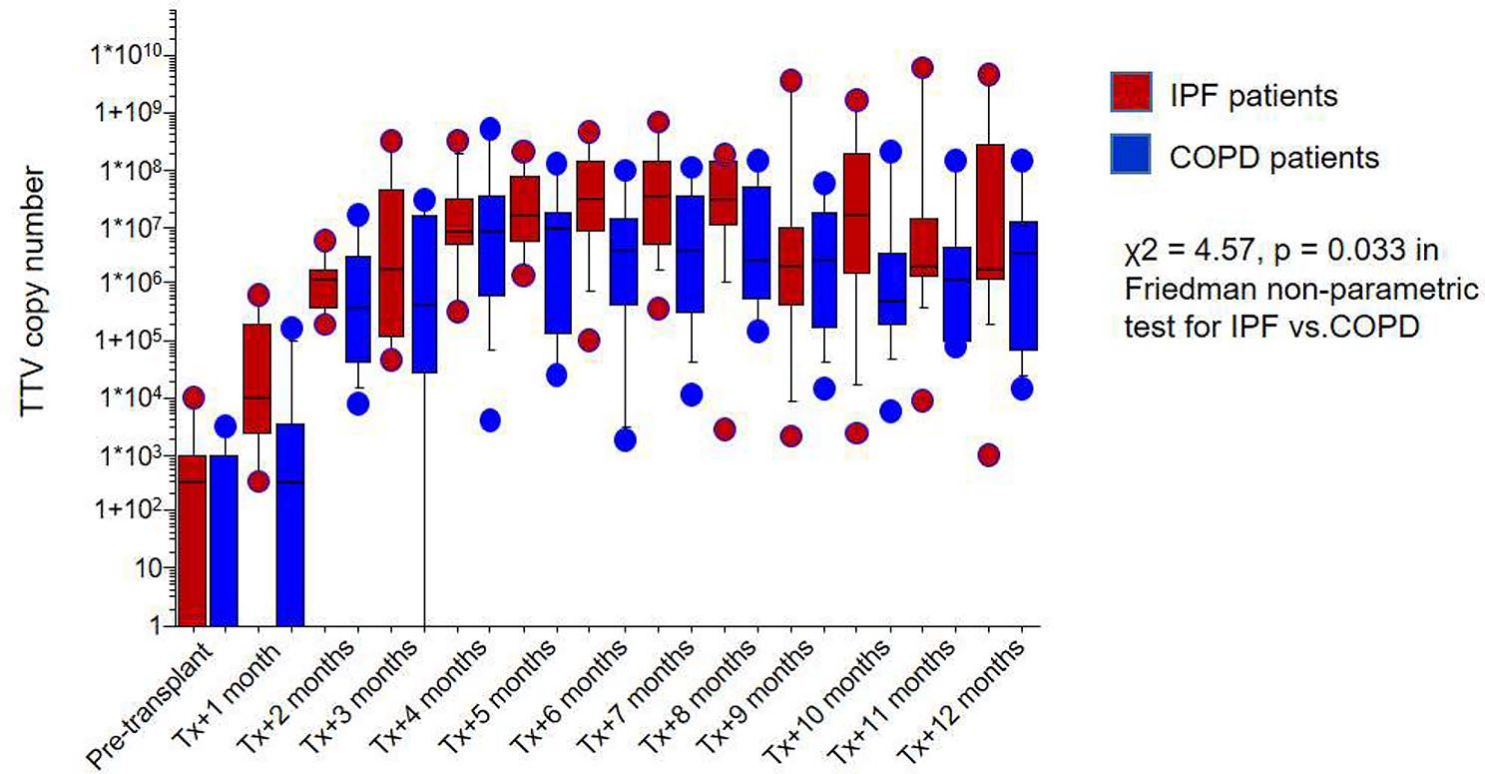
*Torque-Teno Virus* copy number in patients with IPF and COPD. . Increased copies of *Torque-Teno Virus* after lung transplantation in patients with IPF compared to patients with COPD as an underlying disease

The cause of death in LTx recipients with IPF was sepsis and consecutive multi-organ failure in 34/49 (69%) of the patients in that subgroup). To further corroborate this hypothesis, we re-analyzed the *Torque-teno virus* (TTV) load as a possible endogenous marker to reflect immune function in selected patients from our cohort [24]. Interestingly, we found higher TTV-DNA levels after lung transplantation in patients with IPF compared to COPD patients (X^2^ = 4.57, *p* = 0.033; Fig. 5). The incidence of CMV reactivation was similar in both groups, COPD and IPF patients (42% vs. 36%). While there was a higher percentage of CMV reactivations in IPF patients with donor organs ≥ 55 years, this did not reach statistical significance (55% vs. 33%, *p* = 0.16).

## Discussion

Selecting the best donor for a patient remains a challenge in lung transplantation [9]. In our lung transplant recipient cohort, a donor age of ≥ 55 years was associated with impaired organ function and reduced survival in patients with IPF.

### Donor and recipient age

Due to the general shortage of organs, the possibility of an expansion of the donor pool using older organs has been analysed in several studies [14, 15, 27–29]. The results are controversial, and the study populations are mostly heterogeneous. While some studies report comparable short-term and long-term outcome in older donor lungs, Sommer et al. found that spirometry findings after transplant were better in emphysema recipients than in IPF recipients with donor lungs > 70 years of age [28]. Data regarding the differential outcome of patients depending on the underlying pulmonary disease, specifically in patients with IPF is scarce. In this context, the most striking finding in our cohort was that donor age has an impact on severe PGD as a short-term complication, and reduced survival as a long-term consequence in patients with IPF. In contrast to recipients with IPF, the transplantation of a donor organ ≥ 55 years of age for patients with underlying COPD did not affect the incidence of PGD or long-term survival. This is in accordance with another study from Shigemura and colleagues, where COPD patients, which comprised the majority of the cohort, had a similar outcome with organs > 55 years of age [15]. One might speculate that underlying immunological alterations [17] and/or an accelerated ageing process in patients suffering from IPF may increase the susceptibility to infections and, that these patients become even more vulnerable to infections if also the transplanted organ (and the immune cells within) are older. The incidence of CLAD was not affected by donor or recipient age, similar to the results from other studies [27, 30].

While evaluating the importance of donor age depending on the underlying lung disease, we also analysed surgical factors that might impact the study’s results. Single lung transplantation may be a risk factor for increased incidence of post-transplant infection due to bacteria in the remaining native lung. However, there was no statistically significant difference in survival in either group.

ECMO is a significant risk factor for PGD and may also have contributed to the high incidence of PGD in patients with IPF compared to patients with COPD. On the other hand, 58% (25/43) of the donors of the ECMO patients in the IPF group were < 55 years old and there was no statistically significant difference in overall survival with and without ECMO in IPF patients.

### Lung fibrosis and immunological response

Telomere-related gene mutations have a high prevalence in patients with IPF [31] leading to accelerated ageing of the lung. Over the last decade, shorter telomere length has been associated with impaired immune function [32] and a worse outcome after LTx [33]. Transplant recipients are more susceptible to neutropenia [34] under immunosuppressive medication and more prone to lower airway infections. Older donor organs do not have the same self-repair mechanisms compared to their younger counterparts mainly due to endothelial senescence [35]. They additionally have reduced autophagy [18] and impaired pulmonary endothelial protective mechanisms [35, 36]. All of this results in an increased vulnerability to infection, mitigated DNA repair mechanisms, deficits in cell repair and regeneration and thus attenuated parenchymal healing [37].

Most likely, both conditions, the older age of donor organs and the presence of fibrosis as a surrogate of accelerated aging in the recipient [17] are responsible for the observed unfavourable outcome. The hypothesis of aging and immunological processes underlying fibrosis might explain the reduced survival after the transplantation of an older organ with potentially reduced regeneration abilities. This is further supported by the fact that sepsis was the leading cause of death in our cohort in patients with fibrotic lung diseases and older donor age.

Over the last decade, *Torque-Teno Virus* or *Transfusion-transmitted Virus* (TTV) has gained increasing attention as a possible endogenous marker to reflect immune function in solid organ transplantation [24, 38], with higher TTV-DNA copy numbers indicating a more suppressed immune system [20]. There is evidence that the TTV-DNA load may correlate with the level of medical immunosuppression individually after transplantation [20, 39]. In accordance with our hypothesis, there is a significant difference towards higher TTV-DNA levels after lung transplantation in patients with IPF compared to COPD patients, as shown in re-analyzed data from our cohort [24]. However, we were only able to analyze a small sub-cohort of our patients, and the role of TTV load as a marker for immunosuppression is disputable and needs further verification [38], therefore, we cannot draw a definite conclusion here. IPF lung transplant recipients were shown to have an increased incidence of CMV viremia episodes and other CMV complications [40]. However, the association of post-transplant CMV reactivation with TTV copy numbers is controversial [41, 42]. While there was no statistically significant increase in CMV reactivations in patients with IPF compared to patients with COPD in our cohort, this might be due to small numbers.

Yet, one may carefully hypothesize that dysregulation of the immune response in patients with IPF, and potentially in patients with fibrotic lung diseases in general, might impact short- and long-term outcomes after LTx. With these data, we cannot analyse the impact of older donor organs on TTV copy numbers in IPF and COPD patients due to small numbers. Ultimately, the combination of a donor organ with age-related impaired regeneration abilities in donor lungs aged ≥ 55 years, may contribute to reduced survival in these patients.

### Limitations of the study

This a retrospective single-centre study covering nearly 20 years. Due to the retrospective nature of our study, older donors who have not been used for transplantation, were not considered. The observations made in this study may therefore be affected by a selection bias. Despite the long analysis period, the immunosuppressive regime for all patients was determined by our outpatient centre and did not significantly change over time. Changes in surgical technique and ECMO management certainly improved ever since which may have influenced patient outcome.

Unfortunately, data on telomere length was not available for the IPF patients in our cohort, therefore we could not further analyse the hypothesis of accelerated aging and reduced immune function after LTx due to shorter telomeres.

Only two subgroups, IPF and COPD patients, were analysed in depth and the overall cohort is heterogeneous. While the effect of donor age might be relevant for patients with underlying fibrotic lung disease in general, not just with IPF (data not shown), more studies are needed that analyse subgroups of pulmonary diseases leading to transplantation differentially.

Lastly, TTV-DNA levels were taken from a small sub-cohort of IPF and COPD patients. More detailed studies with higher patient numbers are warranted to further elucidate the impact on immune function and the association with immunosuppression drug levels. Although some hypotheses support an altered immune function in IPF patients, potentially contributing to reduced survival in IPF recipients with older donor organs, our data is not sufficient to draw a definite conclusion here. Moreover, due to small numbers, TTV DNA levels in IPF/COPD recipients with donors ≥ 55 years of age cannot be sufficiently evaluated in this study.

## Conclusions

In lung transplant recipients with IPF, a donor ≥ 55 years of age was associated with a higher incidence of primary graft dysfunction. Moreover, it was revealed as an independent risk factor for reduced survival in the whole cohort and in patients with IPF specifically. Thus, precise recipient selection is crucial. Although we may only hypothesise an altered immune function in selected recipients with IPF, from our viewpoint, older donor organs should be carefully evaluated. Since most studies are based on a heterogeneous recipient cohort, further studies are needed to analyse the impact of the underlying pulmonary disease, as this may be a relevant factor for postoperative graft function and survival.

## Electronic supplementary material

Below is the link to the electronic supplementary material.

Supplementary Material 1

## Data Availability

The datasets analysed during the current study are available from the corresponding author on reasonable request.

## Abbreviations

AAT: Alpha-1 Antitrypsin
AHT: Arterial Hypertension
BMI: Body Mass Index
CHD: Coronary Heart Disease
COPD: Chronic Obstructive Pulmonary Diseases
CLAD: Chronic Lung Allograft Dysfunction
DLTx: Double Lung Transplantation
DM: Diabetes Mellitus
EAA: Exogenous Allergic Alveolitis
IPF: Idiopathic Pulmonary Fibrosis
LTx: Lung Transplantation
PGD: Primary Graft Dysfunction
PGD2/3: Primary Graft Dysfunction Grade 2 or 3
SLTx: Single Lung Transplantation
TTV: Torque-Teno Virus

## References

[1] Bos S, Vos R, Van Raemdonck DE, Verleden GM. Survival in adult lung transplantation: where are we in 2020? Curr Opin Organ Transpl. 2020;25(3):268–73. 10.1097/MOT.0000000000000753 32332197

[2] Jungraithmayr W, Yamada Y, Haberecker M, Breuer E, Schuurmans M, Dubs L, et al. CD26 as a target against fibrous formation in chronic airway rejection lesions. Life Sci. 2021;278:119496. 33894269 10.1016/j.lfs.2021.119496

[3] Young KA, Dilling DF. The future of lung transplantation. Chest. 2019;155(3):465–73. 30171860 10.1016/j.chest.2018.08.1036 PMC6435913

[4] Yusen RD, Christie JD, Edwards LB, Kucheryavaya AY, Benden C, Dipchand AI, et al. The Registry of the International Society for Heart and Lung Transplantation: Thirtieth Adult Lung and Heart-Lung Transplant Report–2013; focus theme: age. J Heart Lung Transpl. 2013;32(10):965–78. 10.1016/j.healun.2013.08.007 24054805

[5] Khush KK, Cherikh WS, Chambers DC, Harhay MO, Hayes D Jr., Hsich E, et al. The international thoracic Organ Transplant Registry of the International Society for Heart and Lung Transplantation: thirty-sixth adult heart transplantation report – 2019; focus theme: Donor and recipient size match. J Heart Lung Transpl. 2019;38(10):1056–66. 10.1016/j.healun.2019.08.004 PMC6816343 31548031

[6] Mosher CL, Weber JM, Frankel CW, Neely ML, Palmer SM. Risk factors for mortality in lung transplant recipients aged >/=65 years: a retrospective cohort study of 5,815 patients in the scientific registry of transplant recipients. J Heart Lung Transpl. 2021;40(1):42–55. 10.1016/j.healun.2020.10.009 PMC7770611 33208278

[7] Ehrsam JP, Benden C, Seifert B, Opitz I, Schneiter D, Weder W, et al. Lung transplantation in the elderly: influence of age, comorbidities, underlying disease, and extended criteria donor lungs. J Thorac Cardiovasc Surg. 2017;154(6):2135–41. 28823801 10.1016/j.jtcvs.2017.07.032

[8] Moneke I, Ogutur ED, Kalbhenn J, Hettich I, Passlick B, Jungraithmayr W et al. Independent risk factors for an increased incidence of thromboembolism after lung transplantation. J Thromb Thrombolysis 2023;55(2):252–262 10.1007/s11239-022-02748-9 PMC10011327 36495365

[9] Kukreja J, Chen J, Brzezinski M. Redefining marginality: donor lung criteria. Curr Opin Organ Transpl. 2020;25(3):280–4. 10.1097/MOT.0000000000000764 32304425

[10] Pizanis N, Heckmann J, Tsagakis K, Tossios P, Massoudy P, Wendt D, et al. Lung transplantation using donors 55 years and older: is it safe or just a way out of organ shortage? Eur J Cardiothorac Surg. 2010;38(2):192–7. 20227288 10.1016/j.ejcts.2010.01.054

[11] Chambers DC, Zuckermann A, Cherikh WS, Harhay MO, Hayes D Jr., Hsich E, et al. The international thoracic Organ Transplant Registry of the International Society for Heart and Lung Transplantation: 37th adult lung transplantation report – 2020; focus on deceased donor characteristics. J Heart Lung Transpl. 2020;39(10):1016–27. 10.1016/j.healun.2020.07.009 PMC7737221 32782073

[12] Orens JB, Boehler A, de Perrot M, Estenne M, Glanville AR, Keshavjee S, et al. A review of lung transplant donor acceptability criteria. J Heart Lung Transpl. 2003;22(11):1183–200. 10.1016/s1053-2498(03)00096-2 14585380

[13] Jawitz OK, Raman V, Becerra D, Klapper J, Hartwig MG. Factors associated with short- versus long-term survival after lung transplant. J Thorac Cardiovasc Surg. 2022;163(3):853–60. e2. 33168166 10.1016/j.jtcvs.2020.09.097 PMC8024421

[14] Hayes D Jr., Black SM, Tobias JD, Higgins RS, Whitson BA. Influence of donor and recipient age in lung transplantation. J Heart Lung Transpl. 2015;34(1):43–9. 10.1016/j.healun.2014.08.017 25301358

[15] Shigemura N, Horai T, Bhama JK, D’Cunha J, Zaldonis D, Toyoda Y, et al. Lung transplantation with lungs from older donors: recipient and surgical factors affect outcomes. Transplantation. 2014;98(8):903–8. 24825527 10.1097/TP.0000000000000134

[16] Shenderov K, Collins SL, Powell JD, Horton MR. Immune dysregulation as a driver of idiopathic pulmonary fibrosis. J Clin Invest. 2021;131(2). 10.1172/JCI143226 PMC7810481 33463535

[17] Kolahian S, Fernandez IE, Eickelberg O, Hartl D. Immune mechanisms in Pulmonary Fibrosis. Am J Respir Cell Mol Biol. 2016;55(3):309–22. 27149613 10.1165/rcmb.2016-0121TR

[18] Kuwano K, Araya J, Hara H, Minagawa S, Takasaka N, Ito S, et al. Cellular senescence and autophagy in the pathogenesis of chronic obstructive pulmonary disease (COPD) and idiopathic pulmonary fibrosis (IPF). Respir Investig. 2016;54(6):397–406. 27886850 10.1016/j.resinv.2016.03.010

[19] Gulati S, Thannickal VJ. The aging lung and idiopathic pulmonary fibrosis. Am J Med Sci. 2019;357(5):384–9. 31010465 10.1016/j.amjms.2019.02.008

[20] Jaksch P, Kundi M, Gorzer I, Murakozy G, Lambers C, Benazzo A, et al. Torque Teno Virus as a Novel Biomarker Targeting the efficacy of Immunosuppression after Lung Transplantation. J Infect Dis. 2018;218(12):1922–8. 30053048 10.1093/infdis/jiy452

[21] Roberto P, Cinti L, Napoli A, Paesani D, Riveros Cabral RJ, Maggi F, et al. Torque Teno virus (TTV): a gentle spy virus of immune status, predictive marker of seroconversion to COVID-19 vaccine in kidney and lung transplant recipients. J Med Virol. 2023;95(2):e28512. 36661060 10.1002/jmv.28512 PMC10108096

[22] Rahmel A. Eurotransplant Annual report. 2013.

[23] Snell GI, Yusen RD, Weill D, Strueber M, Garrity E, Reed A, et al. Report of the ISHLT Working Group on Primary Lung Graft Dysfunction, part I: definition and grading-A 2016 Consensus Group statement of the International Society for Heart and Lung Transplantation. J Heart Lung Transpl. 2017;36(10):1097–103. 10.1016/j.healun.2017.07.021 28942784

[24] Frye BC, Bierbaum S, Falcone V, Kohler TC, Gasplmayr M, Hettich I, et al. Kinetics of Torque Teno Virus-DNA plasma load predict rejection in lung transplant recipients. Transplantation. 2019;103(4):815–22. 30234787 10.1097/TP.0000000000002436

[25] Martin-Gandul C, Perez-Romero P, Sanchez M, Bernal G, Suarez G, Sobrino M, et al. Determination, validation and standardization of a CMV DNA cut-off value in plasma for preemptive treatment of CMV infection in solid organ transplant recipients at lower risk for CMV infection. J Clin Virol. 2013;56(1):13–8. 23131346 10.1016/j.jcv.2012.09.017

[26] Thomas D, Wilkinson AJ, Succony L, Tsui S. Cytomegalovirus infection following lung transplantation - occurrence, treatment and risk of OB. J Heart Lung Transplantation. 2014;33(4):S180–1.

[27] Renard R, Girault A, Avramenko-Bouvier A, Roussel A, Cerceau P, Pellenc Q, et al. Outcome of lung transplantation using grafts from Donors over 65 years of age. Ann Thorac Surg. 2021;112(4):1142–9. 33171173 10.1016/j.athoracsur.2020.10.018

[28] Sommer W, Ius F, Salman J, Avsar M, Tudorache I, Kuhn C, et al. Survival and spirometry outcomes after lung transplantation from donors aged 70 years and older. J Heart Lung Transpl. 2015;34(10):1325–33. 10.1016/j.healun.2015.06.002 26186805

[29] Somers J, Ruttens D, Verleden SE, Cox B, Stanzi A, Vandermeulen E, et al. A decade of extended-criteria lung donors in a single center: was it justified? Transpl Int. 2015;28(2):170–9. 25266074 10.1111/tri.12470

[30] Sommer W, Franz M, Aburahma K, Saipbaev A, Flothmann K, Yablonski P, et al. Lungs from donors >/=70 years of Age for Transplantation-Do Long-Term outcomes Justify their use? Transpl Int. 2023;36:11071. 37125386 10.3389/ti.2023.11071 PMC10133456

[31] Alder JK, Armanios M. Telomere-mediated lung disease. Physiol Rev. 2022;102(4):1703–20. 35532056 10.1152/physrev.00046.2021 PMC9306791

[32] Wang P, Leung J, Lam A, Lee S, Calabrese DR, Hays SR, et al. Lung transplant recipients with idiopathic pulmonary fibrosis have impaired alloreactive immune responses. J Heart Lung Transpl. 2022;41(5):641–53. 10.1016/j.healun.2021.11.012 PMC9038662 34924263

[33] Newton CA, Kozlitina J, Lines JR, Kaza V, Torres F, Garcia CK. Telomere length in patients with pulmonary fibrosis associated with chronic lung allograft dysfunction and post-lung transplantation survival. J Heart Lung Transpl. 2017;36(8):845–53. 10.1016/j.healun.2017.02.005 PMC5515686 28262440

[34] Tague LK, Scozzi D, Wallendorf M, Gage BF, Krupnick AS, Kreisel D, et al. Lung transplant outcomes are influenced by severity of neutropenia and granulocyte colony-stimulating factor treatment. Am J Transpl. 2020;20(1):250–61. 10.1111/ajt.15581 PMC6940547 31452317

[35] Schneider JL, Rowe JH, Garcia-de-Alba C, Kim CF, Sharpe AH, Haigis MC. The aging lung: physiology, disease, and immunity. Cell. 2021;184(8):1990–2019. 33811810 10.1016/j.cell.2021.03.005 PMC8052295

[36] Bustos ML, Huleihel L, Kapetanaki MG, Lino-Cardenas CL, Mroz L, Ellis BM, et al. Aging mesenchymal stem cells fail to protect because of impaired migration and antiinflammatory response. Am J Respir Crit Care Med. 2014;189(7):787–98. 24559482 10.1164/rccm.201306-1043OC PMC4061541

[37] Jane-Wit D, Chun HJ. Mechanisms of dysfunction in senescent pulmonary endothelium. J Gerontol Biol Sci Med Sci. 2012;67(3):236–41. 10.1093/gerona/glr248 PMC3297765 22396472

[38] Rezahosseini O, Drabe CH, Sorensen SS, Rasmussen A, Perch M, Ostrowski SR, et al. Torque-Teno virus viral load as a potential endogenous marker of immune function in solid organ transplantation. Transpl Rev (Orlando). 2019;33(3):137–44. 10.1016/j.trre.2019.03.004 30981537

[39] Beland K, Dore-Nguyen M, Gagne MJ, Patey N, Brassard J, Alvarez F, et al. Torque Teno virus load as a biomarker of immunosuppression? New hopes and insights. J Infect Dis. 2014;210(4):668–70. 24688072 10.1093/infdis/jiu210

[40] Popescu I, Mannem H, Winters SA, Hoji A, Silveira F, McNally E, et al. Impaired cytomegalovirus immunity in idiopathic pulmonary fibrosis lung transplant recipients with short telomeres. Am J Respir Crit Care Med. 2019;199(3):362–76. 30088779 10.1164/rccm.201805-0825OC PMC6363970

[41] Hirji A, Mabilangan C, Halloran K, Duan QM, Lien DC, Varughese R, et al. Torque Teno Virus does not predict cytomegalovirus infection Post-lung Transplantation. J Heart Lung Transplantation. 2021;40(4):S337–8.

[42] Maggi F, Focosi D, Statzu M, Bianco G, Costa C, Macera L, et al. Early post-transplant Torquetenovirus Viremia predicts Cytomegalovirus Reactivations in solid organ transplant recipients. Sci Rep. 2018;8(1):15490. 30341363 10.1038/s41598-018-33909-7 PMC6195516

