# Supplementary material for: Donor age over 55 is associated with worse outcome in lung transplant recipients with idiopathic pulmonary fibrosis
Source: BMC Pulm Med. 2024 Oct 9;24:499. doi: 10.1186/s12890-024-03317-x (PMC11465681; doi:10.1186/s12890-024-03317-x)
Supplement: Supplementary file 1 — Supplementary Material 1 [file 12890_2024_3317_MOESM1_ESM.docx]

**Supplementary figure S1:** Patient selection diagram

**Supplementary figure S1:** Patient selection diagram.

**Supplementary Figure S2: Distribution of pulmonary diseases leading to transplantation**

**Supplementary figure S2:** Underlying pulmonary diseases leading to lung transplantation. COPD = *chronic obstructive pulmonary disease*, IPF = idiopathic pulmonary fibrosis, CF= Cystic Fibrosis, EAA = exogenous allergic alveolitis, AAT = alpha-1 antitrypsin deficiency.

**Supplementary figure S3: Impact of a donor age ≥ 60 and ≥ 65 in the whole cohort and in patients with IPF**

**a b**


**c d**

**e f**

**g h**

**Figure S3**: a-d: Kaplan-Meier Analysis of survival in donors ≥60 and ≥65 years of age and incidence of primary graft dysfunction grade 2 or 3 PGD2/3. E-h: Kaplan-Meier Analysis of survival after for patients with IPF for donors and recipients ≥60 and ≥65 years of age. e-h: incidence of primary graft dysfunction grade 2 or 3 PGD2/3. The log-rank test was used for the comparison of the survival curves.
